# Automated analysis of retinal imaging using machine learning techniques for computer vision

**DOI:** 10.12688/f1000research.8996.2

**Published:** 2017-06-22

**Authors:** Jeffrey De Fauw, Pearse Keane, Nenad Tomasev, Daniel Visentin, George van den Driessche, Mike Johnson, Cian O Hughes, Carlton Chu, Joseph Ledsam, Trevor Back, Tunde Peto, Geraint Rees, Hugh Montgomery, Rosalind Raine, Olaf Ronneberger, Julien Cornebise

**Affiliations:** 1 DeepMind, London, EC4A 3TW, UK; 2 Moorfields Eye Hospital NHS Foundation Trust, London, EC1V 2PD, UK; 3 Alexandra House University College London, Bloomsbury Campus, London, WC1N 3AR, UK; 4 Department of Applied Heath Research, University College London, London, WC1E 7HB, UK; 5 Institute of Sport, Exercise and Health, London, W1T 7HA, UK

**Keywords:** Optical Coherence Tomography, machine learning, artificial intelligence, diabetic retinopathy, neovascular age-related macular degeneration, ophthalmology, retina

## Abstract

There are almost two million people in the United Kingdom living with sight loss, including around 360,000 people who are registered as blind or partially sighted. Sight threatening diseases, such as diabetic retinopathy and age related macular degeneration have contributed to the 40% increase in outpatient attendances in the last decade but are amenable to early detection and monitoring. With early and appropriate intervention, blindness may be prevented in many cases.

Ophthalmic imaging provides a way to diagnose and objectively assess the progression of a number of pathologies including neovascular (“wet”) age-related macular degeneration (wet AMD) and diabetic retinopathy. Two methods of imaging are commonly used: digital photographs of the fundus (the ‘back’ of the eye) and Optical Coherence Tomography (OCT, a modality that uses light waves in a similar way to how ultrasound uses sound waves). Changes in population demographics and expectations and the changing pattern of chronic diseases creates a rising demand for such imaging. Meanwhile, interrogation of such images is time consuming, costly, and prone to human error. The application of novel analysis methods may provide a solution to these challenges.

This research will focus on applying novel machine learning algorithms to automatic analysis of both digital fundus photographs and OCT in Moorfields Eye Hospital NHS Foundation Trust patients.

Through analysis of the images used in ophthalmology, along with relevant clinical and demographic information, DeepMind Health will investigate the feasibility of automated grading of digital fundus photographs and OCT and provide novel quantitative measures for specific disease features and for monitoring the therapeutic success.

## Background

Age-related macular degeneration (AMD) is a degenerative retinal disease that can cause irreversible visual loss ( Bressler, 2004). It is the leading cause of blindness in Europe and North America and accounts for over half of partially sighted or legally blind certifications in the UK ( Bunce *et al.*, 2010). Neovascular (“wet”) AMD is an advanced form of macular degeneration that historically has accounted for the majority of vision loss related to AMD. It is characterised by abnormal blood vessel growth that can result in hemorrhage, fluid exudation and fibrosis, and thus to local macular damage and ultimately vision loss ( Owen *et al.*, 2012).

Diabetic retinopathy (DR) is the leading cause of blindness in working age populations in the developed world ( Cheung *et al.*, 2010). It is estimated that up to 50% of people with proliferative DR (characterised by neovascularisation) who do not receive timely treatment will become legally blind within 5 years ( Shaw *et al.*, 2010). Although up to 98% of severe visual loss due to DR can be prevented with early detection and treatment, once it has progressed vision loss is often permanent ( Kollias & Ulbig, 2010). Indeed, 4,200 people in England every year are at risk of blindness caused by diabetic retinopathy and there are 1,280 new cases of blindness caused by diabetic retinopathy ( Scanlon, 2008).

To diagnose these conditions and monitor their progression (and response to treatment) the presence and precise location of the lesions must be determined. Two imaging modalities are commonly used for this purpose: digital photographs of the fundus (the ‘back’ of the eye) and Optical Coherence Tomography (OCT, a modality that uses light waves in a similar way to how ultrasound uses sound waves) ( Huang *et al.*, 1991).

Clinics such as these have generated very large datasets of both digital fundus and OCT images. They are also very busy; ophthalmology clinics often have long waits and average times to urgent first treatments can be greater than two weeks in the busiest clinics.

Machine learning algorithms make use of the rich, varied datasets to find high dimensional interactions between multiple data points ( Murphy, 2012). Most machine learning methods can be thought of as a form of statistics. Some algorithms look for patterns in the data, identifying subgroups within the full sample (known as clustering). Others rely on specific clinical information against which the algorithm compares its predictions and adjusts accordingly (supervised learning).

There have been significant recent advances in the field of machine learning demonstrating algorithms able to learn how to accomplish tasks without instruction ( Mnih *et al.*, 2015; Silver *et al.*, 2016). Recent healthcare applications of such algorithms have shed light on complex genetic interactions in autism ( Uddin *et al.*, 2014) and monitoring of physiological observations in intensive care ( Clifton *et al.*, 2013).

This study aims to combine traditional statistical methodology and machine learning algorithms to achieve automatic grading and quantitative analysis of both digital fundus photograph and OCT in Moorfields Eye Hospital NHS Foundation Trust patients (London, UK). Moorfields Eye Hospital is the leading provider of eye health services in the UK and a world-class centre of excellence for ophthalmic research and education. Should the research be successful, implementation of the outcomes would improve patient access to treatment and ease pressures on time and resources in ophthalmology clinics.

## Aims and objectives

### Primary objective

#### 1.1

Exploratory study: investigate whether computer algorithms can detect and classify pathological features on eye imaging, including fundus digital photographs and OCT.

### Secondary objectives

If the exploratory study is successful:

#### 2.1

To provide novel image analysis algorithms to identify and quantify specific pathological features in eye imaging, using validated methods and expert clinical consensus.

#### 2.2

To provide quantitative measurements disease progression, severity and to monitor the therapeutic success over time.

## Study design

This is a retrospective, non-interventional exploratory study. Analyses performed in the study will be on fully anonymised (to achieve the primary objective) and pseudonymised (to achieve the secondary objective 2.2) retinal images (including fundus images and OCT). These images will contain no patient identifiable information.

### Inclusion criteria

All patients attending Moorfields Eye Hospital NHS Foundation Trust sites between 01/01/2007 and 31/01/2017, and who had digital retinal imaging (including fundus digital photographs and OCT) as part of their routine clinical care, will be eligible for inclusion in this study.

### Exclusion criteria

Hard copy examinations (i.e. physical photograph copies prior to digital storage) will be ineligible, and will be excluded by the nature of the original service evaluation requests from the Moorfields team. Data from patients who have previously manually requested that their data should not be shared, even for research purposes in anonymised form, and have informed Moorfields Eye Hospital of this, will be ineligible and removed by Moorfields Eye Hospital staff before research begins. This information is recorded by the Moorfields Eye Hospital Information Governance team, who are responsible for ensuring these patients are removed from any data transferred.

### Sample size

Approximately 1 million examinations meet the above criteria.

Most recent machine learning algorithms benefit from large datasets on which to train (tens to hundreds of thousands of data instances ( Silver *et al.*, 2016)). Across all machine learning applications the predictive power (as percentage of data instances correctly classified) of the algorithm depends on the size and quality of the dataset.

The sample size is informed by the existing literature and by DeepMind’s previous work in the field of machine learning ( Mnih *et al.*, 2015; Silver *et al.*, 2016). Today’s most powerful deep neural networks can have millions or billions of parameters, so large amounts of data are needed to automatically infer those parameters during learning. Most problems in the medical domain are highly complex as they arise as an interplay of many clinical, demographic, behavioural and environmental factors that are correlated in non-trivial ways. This is even more true for state-of-the art deep learning methodologies that are expected to give the best results ( Szegedy *et al.*, 2014).

### Data

For all patients meeting inclusion/exclusion criteria the following electronic health record data will be required to complete this project successfully:

(1) Digital Fundus Photographs
(2) Digital OCT images

In addition to image data, the anonymised dataset will contain additional information required to train an algorithm (objective 1.1):

• Demographic information shown to be associated with eye disease. This is because the retina differs by features such as age, as do the likelihoods, manifestations and progression of specific disease states.
• Primary and secondary diagnostic labels describing what pathology is in the image (e.g., wet AMD, diabetic retinopathy) and the associated severity (e.g., grade of retinopathy/maculopathy in diabetic retinopathy).
• Treatment information describing aspects of management alongside pathology information and clinical data such as visual acuity.
• Model of imaging device.

A second dataset will be pseudonymised to allow further investigation of disease progression and treatment effects. This will include the above information, and add temporal information allowing pseudonymised data to be joined over time with knowledge of the elapsed time between each:

• Time elapsed between each data point and the first visit in weeks (Sunday to Sunday).
• The dates of each scan will be removed so it will be impossible to identify an individual date or year of patient’s birth.

The anonymisation and pseudonymisation procedures adopted will remove any information not specified to further avoid transfer of patient identifiable information. All anonymisation and pseudonymisation will be formally checked by Moorfields Eye Hospital staff before transfer.

### Algorithm development

In order to develop the algorithm, DeepMind will work with the eye images, split by the known diagnoses, using machine learning and Artificial Intelligence techniques including but not limited to: supervised and semi-supervised convolutional neural networks, recurrent neural networks, unsupervised clustering, reinforcement learning ( Murphy, 2012).

For a selection of images additional manual labels will be produced by experts to allow investigation of the clinical benefit that can be achieved through the use of machine learning in eye imaging. Pathological and anatomical features will be annotated by trained graders at Moorfields Eye Hospital and overseen by a consultant ophthalmologist with over 10 years of experience.

### Statistical analysis

Descriptive statistics will be used to describe specific pathological features extracted by the model on retinal images, both continuous and categorical (e.g. presence and size of an abnormality), and to compare them to an expert reference. This will include investigating accuracy, sensitivity and specificity of any models developed against a consensus gold standard. A formal power calculation will be used to inform this section of the work.

In addition we will analyse the outcomes against recent Moorfields audit data on human performance to further understand the potential impacts of the model in clinical practice.

## Data protection

### Anonymisation and pseudonymisation

This study requires existing retrospective data only; no prospective data are needed nor will be collected from patients, hospitals or healthcare workers. No direct patient contact will occur and necessary data will be anonymised or pseudonymised from this source dataset.

Anonymisation and pseudonymisation of all image files and clinical information is performed and validated by Moorfields Eye Hospital staff at Moorfields Eye Hospital. No patient identifiable data will be transferred to DeepMind.

During validation of the anonymisation procedure it was noted that the current anonymisation tool in use at Moorfields had the potential to leave information that may identify the patient in a small number of datasets. DeepMind collaborators worked with Moorfields Eye Hospital IT team to develop an anonymisation script that reliably deletes all of these information in a second step. This new script will be used in the project and will be run by Moorfields Eye Hospital NHS Foundation Trust staff at Moorfields Eye Hospital. DeepMind will not have access to patient identifiable information at any time.

### Data storage

DeepMind Health has developed and established a state-of-the-art secure patient information handling service utilising Common Criteria EAL4 compliant firewalls and on-disk encryption (using Advanced Encryption Standard with a 256-bit key) of all research data, all housed within an ISO 27001 compliant data centre. After anonymisation data will be transferred to our London, UK data centre. This data handling facility conforms to NHS HSCIC Information Governance Statement of Compliance Toolkit (assessed at level 3).

Access will be granted by the custodian of data and no other members of the team. Only those working directly on the data in a research capacity will have access.

### Data destruction

The data sharing agreement between DeepMind and Moorfields Eye Hospital NHS Foundation Trust lasts for 5 years. After this period the agreement will be reviewed should future work seek to build on this project. After the data sharing agreement expires all data used in the study will be destroyed. No modification will be made based on the data after destruction.

Data destruction will involve the deletion of the encryption/decryption keys for all project volumes, and 8-way random data write to all physical disks within the DeepMind Health data infrastructure. A certificate of destruction will be provided to the Trust.

The algorithms developed during the study will not be destroyed. DeepMind Health knows of no way to recreate the patient images transferred from the algorithms developed. No patient identifiable data will be included in the algorithms.

## Ethical considerations

### Ethical approvals

The research on anonymised data received formal approval from the Moorfields Eye Hospital research and development office, responsible for approving studies working with anonymised data, on 16 Oct 2015 (reference 15/050).

The research on temporally linked data received formal Research Ethics Committee approval on 03 Jun 2016 (reference 16/EE/0253) and subsequently was approved by the Health Research Authority.

### Consent

No patient will be approached directly and this work will include no direct patient contact. Only anonymised or pseudonymised retrospective data collected as part of routine clinical care are included. No patient identifiable data will be collected as part of this study. In such cases the ICO code of practice states that explicit consent is not generally required ( ICO, 2012).

To ensure patients are adequately informed both Moorfields and DeepMind have information on their website directly relating to the work, and a patient engagement event was held and live-streamed over the internet to provide more information to patients.

### Adverse events

The project is non-interventional and does not involve any direct patient contact. We do not anticipate any change to patient management. For pseudonymised data should expert assessment during model validation highlight a clinical error this will be raised to the appropriate clinical team. The primary point of call for adverse events should they arise will be the Trust information governance lead, and the responsible clinical team at Moorfields Eye Hospital will be notified.

### Monitoring

The study will be monitored both internally and externally. Internally DeepMind managers (TB, JL) will oversee and monitor progress on a day-to-day basis, ensuring the protocol is adhered to and no compliance issues arise.

Clinical and methodological experts (PK, GR, RR) are working with DeepMind to further oversee the ethical, clinical and methodological considerations of the project and will advise on at least a weekly basis to ensure no deviation from the described protocol.

Externally the information governance team at the Moorfields Eye Hospital will be consulted before commencing data collection, and weekly thereafter to ensure no deviation from the described protocol.

### Access

DeepMind has access to the required data to support the research aims of this study. To ensure compliance with the common law principle of data confidentiality, DeepMind will only receive anonymised or pseudonymised data from the Trust. DeepMind works with Moorfields Eye Hospital to ensure accuracy and clarity in the data to allow useful and consistent interpretation at all times.

## Dissemination

The results will be disseminated through normal academic channels, initially focusing on conference proceedings and the indexed peer reviewed literature relevant to the fields of machine learning, artificial intelligence, ophthalmology and clinical research. Patient and Public Involvement representatives will be involved at each stage of the research.

## Conclusion

We propose an exploratory study and initial testing of machine learning algorithms to analyse and quantify pathological and anatomical features in eye images. The results will be compared to expert annotations.

Should development be successful we plan to submit additional applications for permission to refine the model if required and ultimately investigate the performance in a clinical implementation setting.
