# Supplementary material for: Automated analysis of retinal imaging using machine learning techniques for computer vision
Source: F1000Res. 2017 Jun 22;5:1573. Originally published 2016 Jul 5. [Version 2] doi: 10.12688/f1000research.8996.2 (PMC5082593; doi:10.12688/f1000research.8996.2)
Supplement: Peer review history

# Peer review: Referee response for version 1

**DOI:** 10.5256/f1000research.9679.r14781

**Published:** 2016-10-10

**Authors:** Sandrine Zweifel

**Affiliations:** 1 Department of Ophthalmology, University Hospital Zürich, Zürich, Switzerland

Thank you very much for the opportunity to review the manuscript by De Fauw and co-workers. The topic is of great interest. Moorefields recently announced their research partnership with DeepMind Health. In this manuscript the project plan is detailed. One million examinations (digital fundus photographs and OCT scans) which met the inclusion criteria will be analysed using machine learning and Artificial Intelligence techniques.

 The authors did not specify why they did not include angiography as an additional imaging modality which is usually used at baseline or at follow-up exams in patients with AMD and diabetic retinopathy. Please add this information.

 Since data security is an important issue to be discussed especially when evaluating such large data sets the authors need to provide more information regarding patient consent. Does everyone who is examined at Moorefields Eye Hospital give a general consent for evaluating their data? Patients are not specifically informed about the project with DeepMind Health, aren't they? So data of patients are only excluded if patients previously (independent of this project) requested that there data should not be shared.

 Although there might be some risk regarding "data security" it outweighs the potential of earlier detection and treatment of million of patients.

I have read this submission. I believe that I have an appropriate level of expertise to confirm that it is of an acceptable scientific standard.

# Peer review: Referee response for version 1

**DOI:** 10.5256/f1000research.9679.r15056

**Published:** 2016-07-19

**Authors:** Yit C. Yang

**Affiliations:** 1 Royal Wolverhampton NHS Trust, Wolverhampton, UK

There is a good introduction of the two main conditions, AMD and DR and their burden on public health.

 Very little information on machine learning to convince the reader that the fundamental processes and pieces of the jigsaw are already available to apply to AMD and DR or in simpler healthcare scenarios.

 How does MEH plan to locate the ID of those who have given instructions for their images NOT to be used?  Is there a database for this specific parameter?

 Good attention to data security and data protection.

 Not very much information on specific data outcomes expectations. Eg how detailed will be grading be of a wet AMD lesion.

 Overall, a novel concept and worth exploring as it will be able to replace human workforce if successful.

I have read this submission. I believe that I have an appropriate level of expertise to confirm that it is of an acceptable scientific standard.
